# An Integrated Backscatter Ultrasound Technique for the Detection of Coronary and Carotid Atherosclerotic Lesions

**DOI:** 10.3390/s150100979

**Published:** 2015-01-07

**Authors:** Masanori Kawasaki

**Affiliations:** Department of Cardiology, Gifu University Graduate School of Medicine, 1-1 Yanagido, Gifu 501-1194, Japan; E-Mail: masanori@ya2.so-net.ne.jp; Tel.: +81-58-230-6523; Fax: +81-58-230-6524

**Keywords:** ultrasound, coronary artery, carotid artery, plaque, tissue, imaging

## Abstract

The instability of carotid and coronary plaques has been reported to be associated with acute coronary syndrome, strokes and other cerebrovascular events. Therefore, recognition of the tissue characteristics of carotid and coronary plaques is important to understand and prevent coronary and cerebral artery disease. Recently, an ultrasound integrated backscatter (IB) technique has been developed. The ultrasound IB power ratio is a function of the difference in acoustic characteristic impedance between the medium and target tissue, and the acoustic characteristic impedance is determined by the density of tissue multiplied by the speed of sound. This concept allows for tissue characterization of carotid and coronary plaques for risk stratification of patients with coronary and cerebral artery disease. Two- and three-dimensional IB color-coded maps for the evaluation of tissue components consist of four major components: fibrous, dense fibrosis, lipid pool and calcification. Although several ultrasound techniques using special mathematical algorithms have been reported, a growing body of literature has shown the reliability and usefulness of the IB technique for the tissue characterization of carotid and coronary plaques. This review summarizes concepts, experimental procedures, image reliability and the application of the IB technique. Furthermore, the IB technique is compared with other techniques.

## 1. Introduction

The tissue characteristics of carotid and coronary plaques have been reported to be associated with stroke and cardiovascular events [1,2]. Stabilization of vulnerable tissue components such as lipid pool and intra-plaque hemorrhaging rather than regression of plaque volume is considered to be of major benefit in the reduction of cerebrovascular events [3]. In 1978, Horie *et al.* examined serial sections of coronary arteries *post mortem* and demonstrated that plaque rupture into the vessel lumen sometimes preceded and caused thrombus formation, which resulted in acute myocardial infarction [4]. In 1992, Mizuno *et al.* performed an angioscopic study *in vivo* and demonstrated that disruption or erosion of vulnerable plaques followed by thrombosis was the most frequent cause of acute coronary syndrome (ACS) [5]. Therefore, tissue characterization of carotid and coronary plaques is important to evaluate the risk of stroke and cardiovascular disease. Conventional echocardiography, especially intravascular ultrasound imaging (IVUS), is widely used to determine calcification and the three layers of the arterial wall. However, differentiation of lipid core from fibrous tissue using echo intensity is difficult [6]. To analyze the tissue characteristics of coronary plaques *in vivo*, several techniques for the tissue characterization of plaques have been developed using mathematical processing of ultrasound signals [7,8]. Nair *et al.* reported an IVUS system for tissue characterization using an autoregressive classification scheme rather than depending on the classic Fourier method [8]. In that study, not only IB values but also other parameters such as frequencies at maximum and minimum power and slope of the regression line of ultrasound backscattered signals were shown to have diagnostic value. On the other hand, intravascular optical coherence tomography (OCT) has been developed to provide high-resolution, cross-sectional images of tissue *in situ* and has an axial resolution of 10 μm and a lateral resolution of 20 μm [9,10]. The OCT images of human coronary atherosclerotic plaques obtained *in vivo* provide more detailed structural information at the site near the vascular lumen than IVUS. However, tissue characterization of the entire coronary artery by OCT is not possible because of the limited penetration depth of light.

Recently, an integrated backscatter (IB) ultrasound technique has been developed [6]. Recent *in vivo* studies demonstrated that ultrasound IB values reflected the structural composition of atherosclerotic lesions of the entire arterial wall and could differentiate the tissue components of atherosclerotic lesions. The purpose of this review is to summarize the ultrasound IB technique and its clinical usefulness for the detection of coronary and carotid atherosclerotic lesions.

## 2. Concepts and Methods

### 2.1. Concepts of Ultrasound IB Technique for the Discrimination of Tissue Components

The ultrasound IB power ratio is a function of the difference in acoustic characteristic impedance between the medium and target tissue, and the acoustic characteristic impedance is determined by the density of tissue multiplied by the speed of sound. The ultrasound IB power ratio is calculated using the following formula:

(1) $$\text{Ultrasound IB power ratio}=10\log\frac{{\left({\text{Z}}_{2}-{\text{Z}}_{1}\right)}^{2}}{{\left({\text{Z}}_{2}+{\text{Z}}_{1}\right)}^{2}}$$

where Z_1_ and Z_2_ are the acoustic characteristic impedance of the medium and target tissue, respectively.

Therefore, when the difference in acoustic characteristic impedance between medium and target is greater, the ultrasound IB power ratio becomes greater. With the ultrasound technique, part of the ultrasound energy returns to the transducer after reflection from tissue, and the ultrasound energy produces an electrical impulse. With the IB ultrasound technique, the IB values for each tissue component are calculated using a fast Fourier transform, and expressed as the average power, measured in decibels (dB), of the frequency component of the backscattered signal from a small volume of tissue. In contrast, the amplitude of the ultrasound signal is converted into gray-scale images without a fast Fourier transform using the conventional ultrasound technique [11].

### 2.2. Tissue Characterization of Carotid Plaques

**Figure 1.**
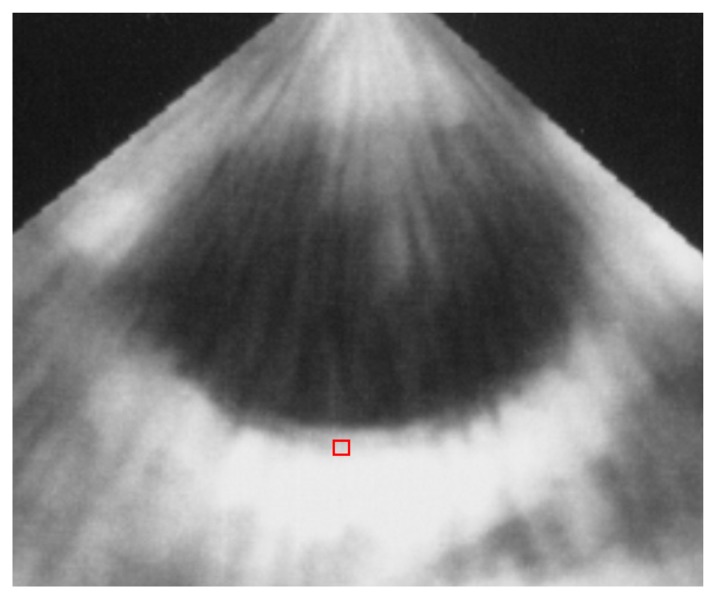
Acquisition of IB value of carotid artery using transthoracic echocardiography. Red square: region-of-interest (11 × 11 pixels).

With the use of transthoracic echocardiography, IB values can be automatically calculated from a region-of-interest (11 × 11 pixels, 0.6 mm × 0.6 mm) set on an IB image using a commercially available system (Sonos 5500 or 7500, Philips Medical Systems, Andover, MA, USA) (Figure 1) [12]. The time gain compensation, which increases amplification of ultrasound signals to correct for increased attenuation of ultrasound signals at greater tissue depths, was set at 0 dB. The lateral gain compensation, which adjusts the gain of one or more scan lines independently, was set at 50 dB. At this setting, the IB value of stainless steel at a distance of 1–2 cm from the transducer was 50 dB, which was within the dynamic range of the system. IB values of the arterial wall were corrected by subtracting the IB values of the vessel lumen or adventitia.

### 2.3. Tissue Characterization of Coronary Plaques

**Figure 2.**
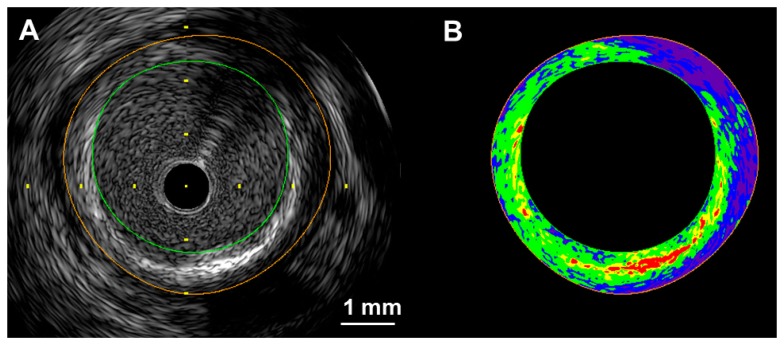
A two-dimensional color-coded map of the coronary arterial plaque. Conventional IVUS images and IB-IVUS color-coded maps were displayed side-by-side on a monitor. Calculation of the relative area of each tissue characteristic was automatically performed by computer software. (**A**) Conventional IVUS image; (**B**) Two-dimensional color-coded map (red: calcification, yellow: dense fibrosis, green: fibrosis, blue and purple: lipid pool). The overall agreement between the classifications made by IB-IVUS and histology (lipid-rich, fibrous and fibrocalcific) was excellent (κ = 0.83, 95% CI: 0.73–0.92) [14].

For the analysis of tissue characteristics using intravascular ultrasound (IVUS), a personal computer equipped with developed custom software was connected to an IVUS imaging system (VISIWAVE, Terumo, Tokyo, Japan) to obtain the ultrasound signal using a 38- and 43-MHz mechanically-rotating IVUS catheter (ViewIT, Terumo) [13]. An analog-to-digital converter was used, which allowed acquisition of signals that were digitized at 400 MHz with 8-bit resolution. In the IVUS analysis, 512 vector lines of ultrasound signal around the circumference were analyzed to calculate the IB values. The tissue IB values were calibrated by subtracting the IB values from the IB value of a stainless steel needle placed at a distance of 1.5 mm from the catheter. IB-IVUS color-coded maps were constructed based on the IB values. Conventional IVUS images and IB-IVUS color-coded maps were immediately displayed side-by-side on a monitor (Figure 2). With a transducer frequency of 38 or 43 MHz, the wavelength was calculated as 36 or 41 μm, respectively (assuming a tissue sound speed of approximately 1560 m/s).

### 2.4. Measurements for Thickness of Fibrous Cap in Coronary Plaques

Recently, intravascular optical coherence tomography (OCT) was shown to provide high-resolution, cross-sectional images of plaques *in situ* with an axial resolution of 10 μm and a lateral resolution of 20 μm [15]. According to a previous pathological and clinical review [16], thin fibrous cap with a large lipid core (thin-cap fibroatheroma) is one of the major criteria for vulnerable plaque that is prone to cause ACS. Therefore, measurement of the thickness of coronary plaques is important to evaluate the risk of cardiovascular disease. To evaluate the accuracy of IB-IVUS for measurement of fibrous cap thickness, the same segments were compared by IB-IVUS and OCT.

### 2.5. Comparison with Virtual Histology Intravascular Ultrasound

Virtual histology IVUS (Virtual Histology Version 1.4, Volcano Corp., Rancho Cordova, CA, USA) images were acquired by a VH-IVUS console with a 20 MHz phased-array catheter and compared with images acquired by IB-IVUS at the same cross-sections. For qualitative comparison, small (0.3 mm × 0.3 mm) region-of-interests (ROIs) were set on the same sites of histological and IVUS images. In quantitative comparison, histological images from cross-sections that were stained with Masson's trichrome were digitized, and the areas that were stained blue were automatically selected by a multipurpose image processor (LUZEX F, Nireco Co., Tokyo, Japan). Then the relative fibrous area (fibrous area/plaque area) was automatically calculated by the LUZEX F system.

## 3. Results

### 3.1. Tissue Characterization of Carotid Plaques

**Figure 3.**
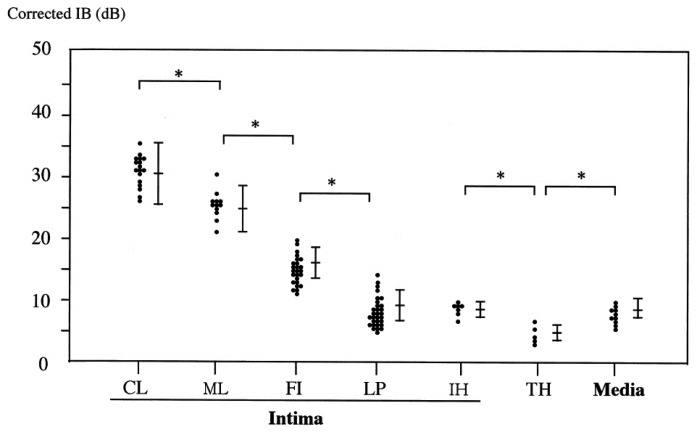
Corrected IB values of various tissue types. The corrected IB values from calcification (CL), mixed lesion (ML), fibrosis (FI), thrombus (TH) and lipid pool (LP), intimal hyperplasia (IH) or media are significantly different from each other. However, there are no significant differences among lipid pool, intimal hyperplasia and media. Mixed lesion: the region in which calcification and fibrosis were mixed. *p < 0.05.

The histological classification of the carotid artery was divided into media and thrombus, lipid pool, intimal hyperplasia, fibrosis, mixed lesion and calcification in the intima (Figure 3) [6].

**Figure 4.**
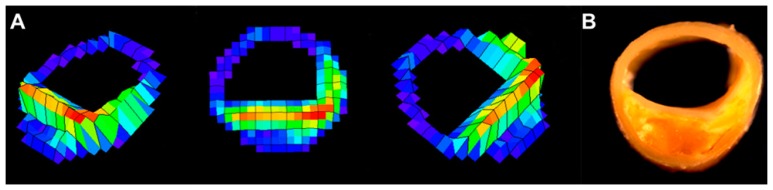
Representative images of carotid plaques. (**A**) Two-dimensional color-coded map of the carotid arterial plaque (red: calcification, yellow and orange: mixed lesion, green: fibrosis, blue and purple: lipid pool); (**B**) Pathological specimen.

Based on the above results, two-dimensional IB color-coded maps of tissue characteristics were constructed (Figure 4).

### 3.2. Tissue Characterization of Coronary Plaques

With the IVUS system, color-coded maps consist of four major components (fibrous [green], dense fibrosis [yellow], lipid pool [blue and purple], calcification [red]) (Figure 2). The overall agreement between the classifications made by IB-IVUS and histology (lipid-rich, fibrous and fibrocalcific) was excellent (κ = 0.83, 95% CI: 0.73–0.92) [14].

### 3.3. Measurements for Thickness of Fibrous Cap in Coronary Plaques

**Figure 5.**
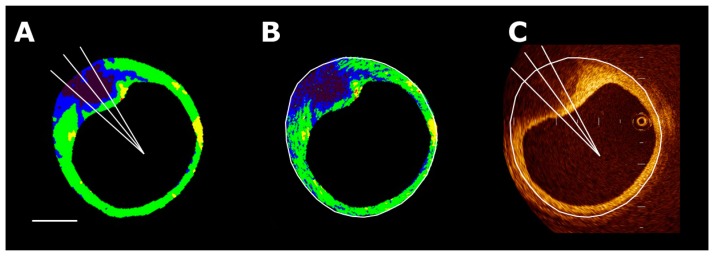
Representative images of coronary plaques. (**A**) IB-IVUS images processed by a smoothing method; (**B**) Original IB-IVUS images; (**C**) Corresponding optical coherence tomography images. Scale bar = 1 mm.

**Figure 6.**
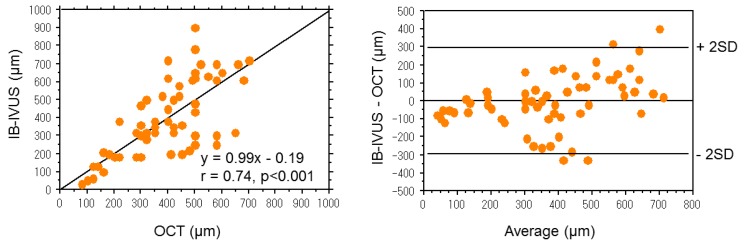
(**Left**) Correlation between the thickness of fibrous cap measured by integrated backscatter intravascular ultrasound and optical coherence tomography; (**Right**) Bland-Altman plot.

The thickness of fibrous cap measured by IB-IVUS was significantly correlated with that measured by OCT in the same coronary segments (Figure 5) [13]. The mean difference between the thickness of fibrous cap measured by IB-IVUS and OCT (IB-IVUS—optical coherence tomography) was −2 ± 147 μm (Figure 6). OCT has a better potential for characterizing tissue components located in the near side from the vessel lumen, whereas IB-IVUS has a better potential for characterizing the tissue components of entire plaques.

### 3.4. Comparison with Virtual Histology Intravascular Ultrasound

**Figure 7.**
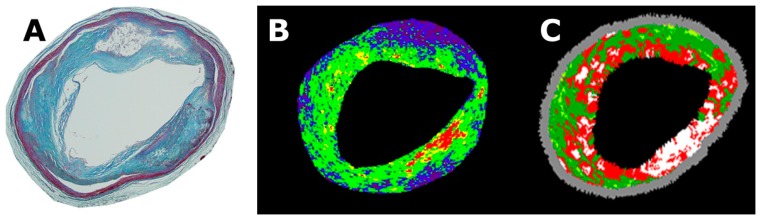
Representative IB-IVUS and VH-IVUS images of the same segment. (**A**) Histological image; (**B**) Corresponding IB-IVUS image; (**C**) Corresponding VH-IVUS image.

For the qualitative comparison, the overall agreement between the histological and IB-IVUS diagnoses was higher (Cohen's κ = 0.81, 95% CI: 0.74–0.90) than that between the histological and VH-IVUS diagnoses (Cohen's κ = 0.30, 95% CI: 0.14–0.41) (Figure 7) [14]. For the quantitative comparison, the % fibrosis area determined by IB-IVUS was significantly correlated with the relative area of fibrosis based on histology (r = 0.67, *p* < 0.001), whereas the % fibrous area and % fibrous area + % fibro-fatty area determined by VH-IVUS were not correlated with the relative area of fibrosis based on histology [14].

## 4. Application of Ultrasound IB Technique for the Evaluation of Plaques

### 4.1. Prediction of Adverse Events after Carotid Artery Stenting

Carotid artery stenting (CAS) has recently emerged as a potential alternative to carotid endarterectomy [17], because it is less invasive and results in a shorter duration of hospitalization. Although many advantages of CAS have been reported, one of its disadvantages is the considerably high incidence of distal emboli during CAS, even though they are subclinical. IB analysis revealed that the relative intra-plaque hemorrhage and lipid pool area in carotid artery stenosis was significantly greater in patients with newly appearing ipsilateral silent ischemic lesions (NISIL) detected by diffusion-weighted magnetic resonance imaging than in patients without NISIL after CAS [18]. Based on receiver-operating characteristic curve analysis, a relative intra-plaque hemorrhage and lipid pool area of 50% measured by IB analysis was found to be the optimal cutoff value for predicting NISIL with a positive predictive value of 76% and negative predictive value of 82%.

### 4.2. Prediction of Adverse Events after Percutaneous Coronary Intervention

A prospective study was performed that determined the optimum cutoff value of relative lipid area in coronary segments without significant stenosis in patients who underwent percutaneous coronary intervention to predict future ACS [19]. Based on receiver-operating characteristic curve analysis, a relative lipid area of >65% measured by IB-IVUS was found to be the optimal cutoff value for predicting ACS with a positive predictive value of 42% and negative predictive value of 98%. Lipid-rich plaques measured by IB-IVUS proved to be an independent morphologic predictor of non-target ischemic events after percutaneous coronary intervention, particularly in those patients with elevated serum C-reactive protein levels [20]. Glagov *et al.* demonstrated that positive remodeling in coronary plaques is a “compensatory process” to maintain the functional size of lumen as a safeguard against narrowing due to atherosclerotic progression with plaque accumulation [21]. Takeuchi *et al.* reported that relative lipid volume measured by IB-IVUS was greater in plaques with positive remodeling than plaques without positive remodeling, and they concluded that there were more lipid-rich components in lesions with positive remodeling than without positive remodeling, which may account for the higher incidence of ACS in those lesions with positive remodeling [22].

### 4.3. Assessment of Arterial Medial Characteristics in Carotid Arteries

In general, atherosclerotic changes consist of two components: atherosis and sclerosis. According to a pathological study, these changes are recognized as: (1) an increase of the intima-media thickness (IMT), which is associated with structural atheromatous changes, and (2) decreased extensibility, which is associated with functional sclerotic changes in elastic and collagen fibers. Ultrasound parameters that can evaluate atherosclerosis include IMT (an index of atheromatous plaque formation) and stiffness β (an index of extensibility of the arterial wall). IMT measurements are widely performed for the detection of atheromatous lesions and an increased IMT is associated with age and coronary risk factors [23]. However, an increased IMT is not always associated with the severity of arterial sclerosis in patients with hypertension [24].

The elasticity of major arteries is also affected by cardiovascular risk factors such as hypertension, hyperlipidemia, diabetes and aging. Stiffness β, which was defined by Hayashi *et al.* was found to be independent of blood pressure in the physiological range and associated with the severity of coronary atherosclerosis [25,26]. An increase in arterial stiffness has been reported as an early sign of atherosclerosis [27]. Therefore, it is important to evaluate arterial sclerosis non-invasively. The IB values of the carotid media were correlated with stiffness β and with the degree of fragmentation of elastic fibers in the carotid media [28].

### 4.4. Evaluation of Effects of Statins by IB Techniques

**Figure 8.**
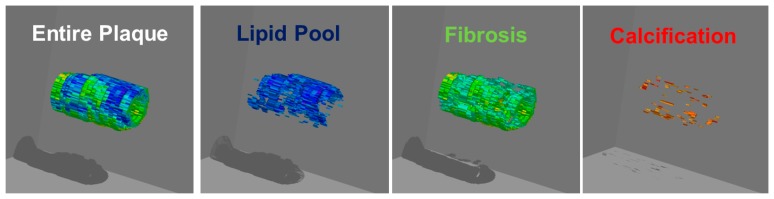
Three-dimensional color-coded maps of the coronary arterial plaques constructed by IB-IVUS.

Several large clinical trials demonstrated that lipid-lowering therapy with 3-hydroxy-3-methylglutaryl coenzyme A reductase inhibitors (statins) reduces cerebrovascular events [29,30]. The IB techniques provide useful clinical information on the effects of statins. The relative lipid volume in carotid plaques measured by IB values significantly decreased in the statin therapy group after 6 months, whereas lipid volume did not change significantly in the diet group [31]. For more comprehensive plaque analysis using IB-IVUS, three-dimensional IB-IVUS offers the potential for quantitative volumetric tissue characterization of coronary atherosclerosis [32] (Figure 8).

Using three-dimensional IB-IVUS, the effect of atorvastatin on coronary plaques was elucidated [32]. The relative lipid volume in coronary plaques measured by IB-IVUS significantly decreased in the statin therapy group after 6 months, whereas lipid volume did not change significantly in the diet group. Otagiri *et al.* investigated the effectiveness of rosuvastatin in patients with ACS using IB-IVUS. They demonstrated that the magnitude of the reduction in relative lipid volume after 6 months of rosuvastatin was significantly correlated with the baseline value (r = −0.498, *p* = 0.024) [33]. This regression was mainly due to a decrease in the lipid component measured by IB-IVUS. Early intervention with rosuvastatin in ACS patients resulted in a significant reduction of the non-culprit plaque after 6 months.

## 5. Discussion

### 5.1. Comparison with Other Techniques

VH-IVUS is one of the ultrasound techniques acquired with a 20 MHz phased-array catheter for tissue characterization of coronary plaques using an autoregressive classification scheme rather than depending on the classic Fourier method [8]. Hiro reported that VH-IVUS images are frequently patchy images of dense-calcium and necrotic core [34]. This is because VH-IVUS uses a classification tree with the eight values in order to discriminate necrotic core, fibrofatty, fibrous, and dense calcium, and the classification tree branches for dense-calcium and necrotic core are very close to each other.

iMap is also one of the intravascular ultrasound techniques for tissue characterization of coronary plaques. The iMap algorithm is based on a neural network theory especially for pattern recognition called the k-nearest neighbor method [35]. It measures a total of 40 values representing how the signal spectrum from the RF segment of interest is similar to each spectrum shape that is specific for necrotic, lipid, fibrotic, or calcified areas from the spectrum shape data-base library, which was previously obtained from cadaver hearts [34]. However, the number of reference points for lipid area is relatively small, and that area is frequently identified as smaller within a plaque than other types of tissue [34]. Yamada *et al.* reported that necrotic tissue area by iMap correlated well with lipid pool area by IB-IVUS, whereas lipidic area by iMap did not correlate with lipid pool area by IB-IVUS, and tissue types classified by iMap generally correlated well with corresponding tissue type by IB-IVUS. However, there was some discrepancy between the two systems [36].

Wavelet analysis is a mathematical model for the detection of lipid pools in coronary plaques reported by Murashige *et al.* [7]. The theoretical basis of wavelet analysis was first developed by Grossmann and Morlet [37]. Wavelet analysis is a time-frequency domain analysis of ultrasound signals. A wavelet is a short segmental waveform of limited duration that has an average value of zero. Wavelet patterns that meet various mathematical criteria have been proposed for comparison and results in many wavelet coefficients, *C*, which are a function of scale and position. The most appropriate *C* of wavelet coefficients for the detection of lipid pool was 0.6 with a sensitivity of 83% and a specificity of 82% [7]. The Wavelet analysis is a unique and different method from IB-IVUS.

Recently, high frequency ultrasound IVUS using 80 MHz without any particular mathematical processing has been proposed [38]. Compared to a 35 MHz ultrasonic image, the 80 MHz image showed superior resolution and contrast with imaging of a rabbit aorta *in vivo*. High frequency IVUS is one of the promising methods for the tissue characterization of coronary plaques.

Intravascular photoacoustic (IVPA) imaging has also been developed and introduced [39]. Combining clinically approved IVUS imaging with IVPA imaging provides supplementing information on atherosclerotic plaque composition. In IVPA imaging, instead of sending acoustic waves into the tissue, a low energy, short laser pulse is emitted into the vessel wall. Absorption of energy from nanosecond duration optical pulses by endogenous tissue or exogenous contrast agents results in a local thermal expansion of tissues which subsequently generates a photoacoustic wave that is detected by an ultrasound transducer [40]. Integrated IVUS/IVPA catheter has been utilized for *in vivo* imaging of human cadavers and live animal models of atherosclerosis [41].

More recently, an integrated IVUS-OCT imaging apparatus, which includes the IVUS and OCT catheter, motor drive unit, and imaging system has been developed [42]. An integrated IVUS-OCT imaging provides high resolution and high penetration depth for a better assessment of vulnerable plaques in *in vivo* animal studies [42]. After solving some potential technical issues, this integrated modality is promising for using in clinical studies.

Current IVUS probes are not optimized for contrast detection because of their design for high-frequency fundamental-mode imaging. However, data from transcutaneous contrast imaging suggests the possibility of utilizing contrast ultrasound for molecular imaging or vasa vasorum assessment to further elucidate atherosclerotic plaque deposition. Despite the promising contrast-to-tissue ratio and vessel imaging capability of this imaging approach, there is a substantial challenge for ultra-broadband contrast-enhanced intravascular ultrasound [43]. The primary limitation is the large frequency span, which is outside of the current bandwidth of commercially available single-frequency transducers. However, Ma *et al.* designed a dual-frequency IVUS transducer to overcome this limitation [44]. The aperture of the receiving element was significantly smaller than the transmission element, enabling matched electrical impedance of both elements and matched acoustic impedance on transmit for increased acoustic deposition in the imaging field of view [44]. The small aperture dual-frequency IVUS transducer is a promising advance in contrast-enhanced IVUS imaging for molecular imaging or vasa vasorum assessment to further elucidate atherosclerotic plaque deposition.

These novel techniques provide supplementing information on atherosclerotic lesions in addition to the information that is detected by IB techniques for the detection of vulnerable plaques.

### 5.2. Technical Considerations of Ultrasound IB Techniques

Fixation and processing of vessels for histopathological examination has been reported to result in a decrease in total vessel cross-sectional area and luminal cross-sectional area, but absolute wall area (total vessel cross-sectional area minus luminal cross-sectional area) did not change in vessels with minimal atherosclerotic narrowing [45,46]. Several studies have documented that formalin fixation does not significantly affect the morphology and quantitative echo characters of plaque tissue in human aorta [12,47].

IB-IVUS occasionally underestimates calcified lesions and overestimates lipid pool behind calcification due to the acoustic shadow derived from calcification. Acoustic shadow caused by calcification hinders precise determination of the tissue characteristics of coronary plaques. However, there have been many cases in which lesions that were classified as lipid pool by IB-IVUS due to the acoustic shadow behind calcification actually included lipid core in the same lesion analyzed by histology (n = 16/21, 76%) [48]. This finding was concordant with previous results demonstrating that necrotic core and fibro-fatty components were located behind calcification (83%–89%) [49]. Since calcification usually originates in lesions with lipid accumulation, the diagnosis of lipid pool by IB-IVUS in lesions behind calcification is usually accurate.

### 5.3. Limitations of the IB Technique

There were a few limitations of the ultrasound method. First, the angle-dependence of the ultrasound signal makes tissue characterization unstable, when lesions are not perpendicular to the axis. Picano *et al.* reported that angular scattering behavior is large in calcified and fibrous tissues, whereas it is slight to nonexistent in normal and fatty plaques [50]. According to that report, although there was no crossover of IB values between fibrous and fibro-fatty tissue within an angle span of 10°, or between fibrous and fatty tissue within an angle span of 14°, this angle-dependence of the ultrasound signal might be partially responsible for the variation of IB values obtained from each tissue component. There was also a report that demonstrated the degree of angle-dependence of 30 MHz ultrasound in detail [51]. In that report, the angle-dependence of 30 MHz ultrasound in the arterial intima and media was 1.11 dB/10°. When a 40 MHz catheter was used, the angle-dependence increased in arterial tissue. This angle-dependence of the ultrasound signal may decrease the diagnostic accuracy for differentiating tissue components. Second, calcification is a perfect reflector for ultrasound, causing acoustic shadowing so typical in IVUS images. The ultrasound signals cannot penetrate, or pass through the calcified layer and are reflected back towards the transducer [11]. Therefore, accurate tissue characterization of the areas behind calcification using IB-IVUS was not possible, as with conventional IVUS. Likewise, IB-IVUS cannot diagnose the tissue behind stents, because stents are nearly perfect reflectors causing acoustic shadowing of the ultrasound signal. This may also decrease the diagnostic accuracy for differentiating the tissue components. Third, a guidewire was not used in the process of imaging because the present studies were performed *ex vivo*. Imaging artifacts *in vivo* due to a guidewire may decrease diagnostic accuracy. Finally, detecting thrombus from a single IVUS cross-section was not possible because we usually looked at multiple IVUS images over time for speckling, scintillation, motion and blood flow in the “microchannel” [11]. The analysis of IB values in multiple cross-sections over time is required for the detection of thrombus.

## 6. Conclusions

Ultrasound IB techniques that depend upon differences of acoustic characteristic impedance among various tissue components have been recently established for the tissue characterization of human carotid and coronary arteries. IB echocardiography and IB-IVUS can detect lipid pools and fibrous tissue in atherosclerotic lesions, and the effects of lipid-lowering therapy. The presence of lipid-rich plaques is associated with the incidence of atherosclerotic diseases; therefore, ultrasound IB techniques are useful to detect coronary and carotid atherosclerotic lesions. In addition, IVPA imaging, combined IVUS-OCT imaging and contrast-enhanced IVUS imaging are also promising techniques that provide important supplementing information on atherosclerotic plaque composition.
